# Randomised controlled trial to improve health and reduce substance use in established psychosis (IMPaCT): cost-effectiveness of integrated psychosocial health promotion

**DOI:** 10.1186/s12888-017-1570-1

**Published:** 2017-12-22

**Authors:** Margaret Heslin, Anita Patel, Daniel Stahl, Poonam Gardner-Sood, Manyara Mushore, Shubulade Smith, Kathryn Greenwood, Oluwadamilola Onagbesan, Conan O’Brien, Catherine Fung, Ruth Ohlsen, David Hopkins, Philippa Lowe, Maurice Arbuthnot, Stan Mutatsa, Gill Todd, Anna Kolliakou, John Lally, Brendon Stubbs, Khalida Ismail, Anthony David, Robin Murray, Zerrin Atakan, Fiona Gaughran

**Affiliations:** 1 King’s Health Economics, Institute of Psychiatry, Psychology & Neuroscience at King’s College London, London, UK; 2 Centre for Primary Care and Public Health, Blizard Institute, Barts and The London School of Medicine and Dentistry, Queen Mary University of London, Yvonne Carter Building, 58 Turner Street, London, E1 2AB UK; 3 Institute of Psychiatry, Psychology & Neuroscience at King’s College London, London, UK; 4 London South Bank University, London, UK; 5 R&D department, Sussex Partnership NHS Foundation Trust, Brighton, UK; 6 School of Psychology, University of Sussex, Brighton, UK; 7 Florence Nightingale Faculty of Nursing and Midwifery, King’s College London, London, UK; 8 Division of Ambulatory Care and Local Networks, King’s College Hospital NHS Foundation Trust, London, UK; 9 King’s College London School of Medicine, London, UK; 10 Carer Advisor, London, UK; 11 Service User Advisor, London, UK; 12 City University, London, UK; 13 South London and Maudsley NHS Foundation Trust, London, UK; 14 Physiotherapy Department, South London and Maudsley NHS Foundation Trust, London, UK

**Keywords:** Health promotion, Psychosis, Quality of life, Economic, Cost

## Abstract

**Background:**

There is mounting evidence that people with severe mental illness have unhealthy lifestyles, high rates of cardiovascular and metabolic diseases, and greater risk of early mortality. This study aimed to assess the cost-effectiveness of a health promotion intervention seeking to improve physical health and reduce substance use in people with psychosis.

**Methods:**

Participants with a psychotic disorder, aged 18-65 years old and registered on an enhanced care approach programme or equivalent were recruited from community mental health teams in six mental health trusts in England. Participants were randomisation to either standard community mental health team care (treatment as usual) or treatment as usual with an integrated health promotion intervention (IMPaCT). Cost-effectiveness and cost-utility analyses from health and social care and societal perspectives were conducted alongside a cluster randomised controlled trial. Total health and social care costs and total societal costs at 12 and 15 months were calculated as well as cost-effectiveness (incremental cost-effectiveness ratios and cost-effectiveness acceptability curves) at 15 months based on quality of life (SF-36 mental and physical health components, primary outcome measures) and quality adjusted life years (QALYs) using two measures, EQ-5D-3 L and SF-36. Data were analysed using bootstrapped regressions with covariates for relevant baseline variables.

**Results:**

At 12-15 months 301 participants had full data needed to be included in the economic evaluation. There were no differences in adjusted health and social care costs (£95, 95% CI -£1410 to £1599) or societal costs (£675, 95% CI -£1039 to £2388) between the intervention and control arms. Similarly, there were no differences between the groups in the SF-36 mental component (−0.80, 95% CI -3.66 to 2.06), SF-36 physical component (−0.68, 95% CI -3.01 to 1.65), QALYs estimated from the SF-36 (−0.00, −0.01 to 0.00) or QALYs estimated from the EQ-5D-3 L (0.00, 95% CI -0.01 to 0.02).

Cost-effectiveness acceptability curves for all four outcomes and from both cost perspectives indicate that the probability of the health promotion intervention being cost-effective does not exceed 0.4 for willingness to pay thresholds ranging from £0-£50,000.

**Conclusions:**

Alongside no evidence of additional quality of life/clinical benefit, there is also no evidence of cost-effectiveness.

**Trial registration:**

ISRCTN58667926. Date retrospectively registered: 23/04/2010. Recruitment start date: 01/03/2010.

## Background

There is mounting evidence that people with severe mental illness have unhealthy lifestyles [1–4], high rates of cardiovascular and metabolic diseases [5], and greater risk of early mortality [6, 7]. These major health implications inevitably carry substantial economic consequences, both within and outside of the health system [8]. There is an urgent need to address modifiable lifestyle factors to reduce cardiovascular and other diseases associated with morbidity and mortality [2, 4, 9, 10]. There is a particularly urgent need locally, with the levels of cardiometabolic abnormalities in South London [11] among the highest reported in the world [5]. One promising way to achieve this is through increasing staff awareness of their role in achieving this [12].

We developed a new health promotion intervention (HPI) designed to be integrated into routine clinical care and implemented by the patient’s usual care coordinator – the main clinical contact (from one of a number of professional backgrounds) for patients with psychosis receiving secondary mental health services in the UK. We present here the findings from an economic evaluation of this intervention within a cluster randomised controlled trial. To our knowledge there are no other economic evaluations of integrated health promotion interventions for people with psychosis. Economic evaluations of specific, separate interventions [13, 14] suggest greater costs associated with achieving outcome improvements, rather than any clear economic advantages. Thus there remains a need for cost-effective approaches to addressing this issue.

## Methods

### Design and intervention

Full details of the pragmatic multi-centre phase III two-arm cluster RCT trial and findings from its effectiveness study have been described elsewhere [15–17]. Briefly, community care coordinators with a minimum of four patients on their caseload in participating community mental health teams (CMHTs) were approached in a random sequence and invited to participate. After gaining their informed consent to participate, we approached patients on their caseload meeting the inclusion criteria (18-65 years old with a diagnosis of psychotic disorder (ICD-10 F20-29, F31.2, F32.3, F33.3) under the care of a Community Mental Health Team (CMHT) registered on an enhanced level of the Care Approach Programme (CPA) or equivalent). Exclusion criteria are described elsewhere [15] and we did not recruit from first episode services.

After completing baseline assessments on all consenting patients in a care co-ordinator’s caseload, care coordinators were randomised, stratified by borough, using randomisation blocks of random sizes to deliver either treatment as usual (TAU) with an integrated 9 month intensive HPI (IMPaCT therapy) or treatment as usual alone. All care coordinators were provided a one-off information session on mental and physical health issues. All outcome assessments were undertaken by researchers blind to treatment allocation. It was hypothesised that the intervention arm would have better quality of life and health outcomes at 12 month follow-up, and that this would be sustained 3 months after completion of the formal intervention, at 15-months follow up.

The economic evaluation was integrated into the trial and was based on primary data collection within the trial. It focused on costs at 15 months (for the previous 3 months) from two perspectives: health and social care; and societal.

Ethical approval was obtained from the joint South London and Maudsley and the Institute of Psychiatry NHS Ethics Committed (REC Ref no 09/HO80/41).

### Data collection

An adapted version of the Client Service Receipt Inventory (CSRI) [18] was used to measure individual-level resource use. It covered the use of (all-cause) secondary and community-based health and social care services, prescription medication, time off work, and key social security benefits received by participants and carers. It was administered as a retrospective self-report questionnaire-based interview conducted by assessors blind to treatment allocation. It covered the previous 6-month period at baseline and 12 month follow-up, and the previous 3-month period at 15 month follow-up. Data related to delivery of the intervention were recorded by care coordinators using specifically designed proformas.

### Unit costs

Unit costs (see online supplementary material) were applied to individual-level resource use data to calculate total costs. Briefly, unit costs for most hospital and primary care services were obtained from the NHS Reference Costs [19] (inflated to 2011-12 prices using the Hospital and Community Health Services Pay and Prices Index or Retail price index as appropriate [20]) and the Unit Costs of Health and Social Care [20]. Medication unit costs, taken from the British National Formulary [21] were converted into cost per milligram (mg) based on the most cost-efficient pack size, choosing maintenance doses over initial treatment doses and generic formulations over branded ones to obtain conservative estimates. Lost productivity costs were estimated by applying national average wage rates to lost work days (human capital approach) and were capped at 5 days per week.

The cost of the intervention is described in full elsewhere [17]. Briefly, the intervention consisted of four components and we estimated costs for each of these: production of manuals (excluding the development work); training care coordinators; ongoing supervision of care coordinators; and implementation of the intervention by care coordinators to trial participants. The mean cost of the IMPaCT intervention was £226.40. The comparable cost for patients in the TAU arm was £3.52 in relation to the one-off information session provided to all care coordinators.

All costs are reported in pounds sterling (£) at 2011-12 prices. Costs related to the intervention were not discounted since they were incurred within the first year. However, all other costs (and outcomes) related to the 12-15 month assessment period were discounted using a rate of 3.5% [22].

### Outcomes

All outcome measures were administered as interviewer-administered self-report questionnaires at baseline, 12 and 15 month follow-ups. Cost-effectiveness analyses were based on the joint primary outcome measures, the SF-36 mental component score and SF-36 physical component score [23]. Cost-utility analyses were based on QALYs derived from the SF-36 (US version 1) via the SF-6D and the EQ-5D-3 L [24]. Appropriate utility weights were attached to health states for each measure at baseline, 12 and 15 months [25, 26]. QALY gains between 12 months and 15 months were then calculated using the total area under the curve approach with linear interpolation between assessment points [27].

### Analyses

Data were analysed using Stata (version 11) [28]. Participants were analysed according to the group to which they were randomised regardless of intervention compliance. No normalisation was used, and outliers were not adjusted or removed.

Costs and outcomes were compared at baseline, 12 and 15 months and are presented as mean values by arm with standard deviations. Mean differences and 95% confidence intervals (CIs) were obtained by non-parametric bootstrap regressions (ordinary least squares (OLS), 1000 repetitions) to account for the non-normal distribution commonly found in economic data, with adjustment for clustering at the care coordinator level. To provide more relevant treatment-effect estimates [29] (OLS) regressions to calculate mean differences in costs at 12 and 15 months included covariates for the baseline value for the same cost category, baseline SF-36 mental component score, baseline SF-36 physical component score, baseline SF-36 utility and baseline EQ-5D-3 L utility, plus baseline demographic variables expected to be associated with costs (gender, ethnicity, borough). Similarly, comparisons of outcome data included covariates for baseline: SF-36 mental component score, SF-36 physical component score, SF-36 utility and EQ-5D-3 L utility, plus baseline demographic variables expected to be associated with outcome (gender, age, ethnicity, place of birth and borough).

Individual item non-response for the CSRI was minimal given the interview approach taken. Where it occurred, an item cost was imputed using the mean cost for the same item for other users in the same trial arm and at the same assessment point. Where this was not possible, the overall cost component was imputed using the mean cost for the same cost component in the same trial arm at the same assessment point. For medication data, a series of assumptions and imputations were necessary depending on the nature of the missing information, as follows, making use of available data components where possible. If medication name was missing, we applied an average prescription cost (from Department of Health prescription cost analysis (PCA)), accounting for the reported number of days on that medication, and assuming the prescription lasted for 1 month. If number of days on medication was missing, a PCA average item cost for that medication was used, with the assumption that the patient was prescribed that medication just once in that period. If dose was missing, a PCA average item cost was used, assuming each prescription lasted 1 month but accounting for number of days on the medication. If the dose unit was missing, a PCA average item cost was used assuming each prescription lasted 1 month, with an account of the number of days on medication. If dose frequency was missing, a PCA average item cost was used, assuming each prescription lasted 1 month, again accounting for number of days. Finally, if it was unknown whether the medication was administered as a depot, a PCA average item cost was used assuming each prescription lasted 1 month, accounting for the number of days on medication.

The base case analysis was undertaken using cases with available relevant cost and/or outcome data (i.e. excluding those lost to follow-up for the CSRI, EQ-5D-3 L or SF-36 assessments as relevant).

The economic evaluation takes a decision-making approach which ignores statistical significance (of both the clinical and economic outcomes) and instead focusses on the probability of one intervention being cost-effective compared to another in light of the available data. This is the approach recommended over traditional reliance on decision rules regarding statistical significance [30, 31]. Cost-effectiveness and cost-utility analyses were conducted at 15 months to focus on the more pertinent question of whether any effect lasted beyond the end of the intervention, but 12 month cost and outcome data are also reported for information. The economic evaluation examined 8 possible cost-outcome combinations (accounting for the two cost perspectives and four outcomes). Incremental cost-effectiveness ratios (ICERs) were calculated for any combination showing both higher costs and better outcomes in either the intervention group or control group (it is unnecessary to calculate ICERs for any combinations where one group shows both lower costs and better outcomes as it is then considered to ‘dominate’ the other group).

Uncertainty around cost-effectiveness/cost-utility was explored using cost-effectiveness planes and cost-effectiveness acceptability curves (CEACs) based on the net-benefit approach [32]. These curves are an alternative to confidence intervals around ICERs and show the probability that one intervention is cost-effective compared to the other, for a range of values that a decision maker would be willing to pay for an additional unit of an outcome. Net benefits for each participant were calculated using the following formula, where λ is the willingness to pay for one additional unit of outcome: Net benefit = (λ x outcome) - cost.

A series of net benefits were calculated for each individual for a λ range that would include any policy-making perspectives relevant at the time of analysis. After calculating net benefits for each participant for each value of λ, coefficients of differences in net benefits between the trial arms were obtained through a series of bootstrapped linear regressions (1000 repetitions) of group upon net benefit which included the same covariates used for the comparisons of mean costs and outcomes (i.e. baseline value of: the same cost category, SF-36 mental component score; SF-36 physical component score; EQ-5D-3 L utility score; SF-36 utility score; gender; age; ethnicity; place of birth; borough) and an adjustment for clustering by care coordinator. The resulting coefficients were then examined to calculate for each value of λ the proportion of times that the intervention group had a greater net benefit than the control group. These proportions were then plotted to generate CEACs for all eight cost-outcome combinations.

Although the intervention was conducted for 9 months, cost-effective analyses were conducted on the 12-15 month data. This was done for two reasons. Firstly, to allow a broad enough time window to conduct outcome assessments, which was necessary due to the data collection approach needed here. Secondly, a 9-month assessment could misrepresent cost-effectiveness of the intervention if any outcome improvements or cost savings were subsequently not sustained even for 3 months.

### Sensitivity analyses

We conducted four sensitivity analyses to check the robustness of the base case analyses defined above. First, we explored the potential impact of excluding those lost to follow-up. We examined key socio-demographic and clinical characteristics for those included and excluded from the analyses and conducted an intention to treat (ITT) analysis which included those lost to follow-up by imputing missing total costs and outcomes using imputation in STATA [28]. Imputations of costs and outcomes were based on variables which were expected to be associated with costs and outcomes. For cost imputations, these variables were baseline and 12 month values for the: equivalent cost category; SF-36 mental component score; SF-36 physical component score; EQ-5D-3 L utility score; SF-36 utility score; plus gender, ethnicity, borough, age, place of birth and care coordinator. Imputation of outcomes was based on baseline and 12 month values of the: SF-36 mental component score; SF-36 physical component score; EQ-5D-3 L utility score; SF-36 utility score; plus gender, age, ethnicity, age, place of birth and borough, and care coordinator. Secondly, to explore the potential impact of having follow-up interviews conducted outside of the planned assessment window (more than 30 days before or after the follow-up date), we conducted a ‘correct time window’ analysis including only those trial participants whose data were collected within the correct window. Thirdly, to explore the potential impact of insufficient implementation of the IMPaCT Therapy, we conducted a per protocol analysis which included only those intervention arm participants who received the pre-defined minimum of six intervention sessions of at least 30 min duration each. Finally, to explore the potential impact of care coordinator drop out, we conducted analyses which included only those participants whose care coordinator remained the same throughout the study.

For each of these sensitivity analyses, we examined whether conclusions concerning the mean difference in costs or outcomes between the two trial arms differed to those drawn from the base case analyses.

### Patient and public involvement

Service users and carers, with lived experience were involved throughout the study, from applying to funding to managing the steering group, to co-authoring this paper. Focus groups were also run with service users to refine our approach. Additionally a delphi process with service users was used to develop the health promotion intervention.

## Results

One hundred four care coordinators were recruited and randomized. Four hundred six patients from randomized care coordinators were eligible and consented for the trial. Fifty two care coordinators with 213 patients were randomized to the IMPaCT Therapy and 52 care coordinators with 193 patients were randomized to TAU.

Responses rates for the client service receipt inventory were 100% (n405), 79% (n319) and 74% (n301) at baseline, 12 months and 15 months respectively and similar between the intervention and control group. Corresponding response rates for the SF-36 were 99% (n402), 77% (n313) and 73% (n297), and for the EQ-5D-3 L were 100% (n404), 78% (n315) and 74% (n301). All participants had full data on intervention use. There were no notable differences in the baseline characteristics of the sub-samples included in the base case analyses of those with available data against the full sample.

### Resource use

Resource use patterns at 12 and 15 months are described in Tables 1 and 2. These were not compared statistically since the economic evaluation was focused on costs and cost-effectiveness/utility, and to avoid problems associated with multiple testing. The data suggest that both arms were broadly balanced in their use of core services both before and during the study. As would be expected for this group of patients, service use is very broad in both nature and sector, illustrating the complexity of their care provision.

**Table 1:** Resource use at 12 month follow-up (for the previous 6 months)

		Intervention (*n* = 160)			Controls (*n* = 159)		
*Resource*	*Unit*	*Number of users*	*Mean contacts* ^*a*^	*SD*	*Number of users*	*Mean contacts* ^*a*^	*SD*
*Specialist accommodation*							
Supported housing / assisted living	bed day	37	182	1	30	179	12
Sheltered housing	bed day	1	182	–	6	158	60
Hostel / shelter	bed day	4	182	0	5	152	68
*Hospital inpatient*							
Inpatient	bed day	42	182	1	41	173	34
*Hospital outpatient*							
Psychiatric outpatient	visit	13	4	2	6	2	1
Non-psychiatric / general / medical outpatient	visit	14	3	2	16	2	2
Diabetes clinic	visit	11	3	3	9	1	1
Blood tests	visit	79	5	4	69	4	3
Psychiatric day hospital	visit	2	2	1	1	6	–
Non-psychiatric / general / medical day hospital	visit	2	1	0	2	4	4
Day surgery centre	visit	4	2	1	6	1	0
A&E department	visit	22	2	4	19	2	1
X-ray	visit	23	1	1	14	1	<1
Substance misuse clinic	visit	3	10	12	3	7	4
Dietetics	visit	4	2	3	1	1	–
*Community based day services*							
Community based services	visit	70	44	40	63	40	36
*Community based professionals*							
Care coordinator	surgery visit	95	9	7	98	7	7
Care coordinator	home visit	67	9	8	64	8	8
Care coordinator	phone call	32	8	10	29	6	7
Home treatment team	surgery visit	1	1	–	1	1	–
Home treatment team	home visit	10	17	11	3	13	7
Home treatment team	phone call	1	2	–	0	–	–
Crisis resolution team	surgery visit	1	3	–	0	–	–
Crisis resolution team	home visit	1	2	–	0	–	–
Crisis resolution team	phone call	1	2	–	0	–	–
Community psychiatric nurse	surgery visit	1	6	–	2	4	4
Community psychiatric nurse	home visit	2	5	2	3	3	3
Social worker	surgery visit	4	3	3	5	2	1
Social worker	home visit	2	9	4	2	3	2
Psychiatrist	surgery visit	85	2	2	86	2	4
Psychiatrist	home visit	7	5	9	8	6	8
Psychologist	surgery visit	10	11	10	15	11	13
Psychologist	home visit	1	14	–	1	24	–
Psychologist	phone call	0	–	–	1	1	–
Psychotherapist	surgery visit	1	4	–	1	3	–
Counsellor	surgery visit	9	4	5	5	4	2
GP	surgery visit	110	3	3	104	3	4
GP	home visit	1	3	–	2	3	2
GP	phone call	1	1	–	1	1	–
Blood test at GP	surgery visit	38	2	1	44	2	2
Diabetes nurse	surgery visit	9	2	4	6	2	1
Diabetes nurse	phone call	0	–	–	3	1	0
Practice nurse	surgery visit	33	3	3	21	11	39
Practice nurse	home visit	0	–	–	1	6	–
Practice nurse	phone call	1	2	–	0	–	–
District nurse	surgery visit	2	6	6	0	–	–
Occupational therapist	surgery visit	4	6	7	2	6	4
Occupational therapist	home visit	4	22	34	2	3	0
Occupational therapist	phone call	1	2	–	1	2	–
Dietician	surgery visit	3	1	0	6	3	3
Home help	home visit	11	53	52	7	61	59
Meals on wheels	home visit	2	13	16	0	–	–
Pharmacist for advice	surgery visit	16	2	2	14	3	2
Pharmacist for advice	phone call	2	1	0	0	–	–
NHS direct	phone call	0	–	–	2	2	1
Samaritans	phone call	5	79	90	4	24	45
*Medication*		159	–	–	158	–	–

^a^Mean for users only

All quantities are rounded to nearest whole number

**Table 2:** Resource use at 15 month follow-up (for the previous 3 months)

		Intervention (*n* = 152)			Controls (*n* = 149)		
*Resource*	*Unit*	*Number of users*	*Mean contacts* ^*a*^	*SD*	*Number of users*	*Mean contacts* ^*a*^	*SD*
*Specialist accommodation*							
Supported housing / assisted living	bed day	36	90	3	30	90	3
Sheltered housing	bed day	2	70	30	6	91	0
Hostel / shelter	bed day	1	81	–	5	91	0
*Hospital inpatient*							
Inpatient	bed day	39	90	8	41	90	2
*Hospital outpatient*							
Psychiatric outpatient	visit	8	2	1	1	1	–
Non-psychiatric / general / medical outpatient	visit	7	1	<1	10	2	2
Diabetes clinic	visit	4	1	0	6	1	0
Blood tests	visit	63	2	2	56	3	1
Psychiatric day hospital	visit	2	5	1	0	–	–
Non-psychiatric / general / medical day hospital	visit	1	2	–	3	1	0
Day surgery centre	visit	3	1	0	2	1	0
A&E department	visit	15	1	1	15	1	1
X-ray	visit	10	1	0	12	1	1
Substance misuse clinic	visit	2	24	17	1	1	–
Dietetics	visit	2	1	0	2	1	0
*Community based day services*							
Community based services	visit	57	20	19	50	26	25
*Community based professionals*							
Care coordinator	surgery visit	78	5	6	70	4	4
Care coordinator	home visit	59	5	3	52	4	3
Care coordinator	phone call	28	5	6	26	5	5
Home treatment team	surgery visit	2	2	1	1	8	–
Home treatment team	home visit	7	9	8	4	12	13
Crisis resolution team	surgery visit	1	1	–	0	–	–
Crisis resolution team	home visit	1	1	–	1	1	–
Early intervention team	surgery visit	1	36	–	0	–	–
Community psychiatric nurse	surgery visit	6	6	4	13	4	2
Community psychiatric nurse	home visit	2	4	1	3	1	1
Community psychiatric nurse	phone call	3	8	10	4	5	5
Social worker	surgery visit	2	8	6	4	5	5
Social worker	home visit	0	–	–	2	7	7
Social worker	phone call	0	–	–	1	5	–
Psychiatrist	surgery visit	65	1	1	60	1	1
Psychiatrist	home visit	3	5	6	5	4	5
Psychologist	surgery visit	14	6	5	8	5	5
Psychologist	home visit	1	10	–	0	–	–
Psychotherapist	surgery visit	2	10	3	0	–	–
Psychotherapist	home visit	0	–	–	1	1	–
Counsellor	surgery visit	3	2	2	1	2	–
GP	surgery visit	81	3	2	83	2	1
GP	home visit	1	1	–	0	–	–
GP	phone call	1	2	–	1	4	–
Blood test at GP	surgery visit	26	2	5	27	1	1
Diabetes nurse	surgery visit	4	2	2	3	1	1
Diabetes nurse	home visit	1	1	–	0	–	–
Practice nurse	surgery visit	16	2	2	21	2	1
Practice nurse	phone call	1	1	–	0	–	–
District nurse	surgery visit	3	21	34	2	46	62
District nurse	home visit	1	24	–	0	–	–
Occupational therapist	surgery visit	4	12	9	5	5	4
Occupational therapist	home visit	2	13	16	3	12	0
Occupational therapist	phone call	1	3	–	0	–	–
Dietician	surgery visit	1	1	–	6	2	1
Dietician	home visit	0	–	–	1	12	–
Home help	home visit	12	38	52	4	39	37
Meals on wheels	home visit	4	47	33	1	15	–
Pharmacist for advice	surgery visit	6	3	2	8	3	4
Pharmacist for advice	phone call	2	2	1	1	1	–
NHS direct	phone call	2	7	8	5	3	5
Samaritans	phone call	4	36	39	4	15	21
*Medication*		149			145		

^a^Mean for users only

All quantities are rounded to nearest whole number

### Costs and outcomes

We present total costs from the two cost perspectives and sub-totals for the components within these (generally by sector) (Table 3). There were no differences in these sub-totals by trial arm, except that the cost of the intervention was naturally higher in the intervention group given the additional inputs required compared with the control group (adjusted mean difference £311, 95% CI £267 to £355) and costs borne by charities were higher in the intervention group at 12 months (adjusted mean difference £80, 95% CI £9 to £151). Health and social care and lost productivity formed the largest components of total societal costs.

**Table 3:** Costs at baseline, 12 and 15 months (2011/12 prices, all 15 month costs, except the intervention costs, are discounted)

		Intervention *n* = 213			Control *n* = 193		Unadjusted mean difference^d^	95% CI^d^	Adjusted mean difference^e^	95% CI^e^
	*valid n*	*Mean £*	*SD*	*valid n*	*Mean £*	*SD*				
Component Costs at Baseline										
Health & social care excluding intervention^b^	212	10,242	13,374	193	9714	13,767	528	−2953 to 4010	967	−2442 to 4435
Charity^b^	212	83	611	193	80	435	3	−109 to 115	−22	−137 to 94
Lost productivity^b^	212	8755	5964	193	7472	6311	1283	−354 to 2920	456	−894 to 1806
Patient^b^	212	72	433	193	188	188	35	−31 to 102	33	−37 to 104
Benefits^b^	212	2211	1006	193	2009	940	202^a^	13 to 391^a^	127	−70 to 324
Component Costs at 12 month										
Health & social care excluding intervention^b^	160	10,220	12,341	159	10,196	16,987	24	−4219 to 4267	−1596	−5145 to 1954
Charity^b^	160	120	369	159	61	256	60	−6 to 125	80^a^	9 to 151^a^
Lost productivity^b^	160	8882	5998	159	7707	6333	1174	−317 to 2665	1038	−367 to 2443
Patient^b^	160	84	369	159	53	300	31	−38 to 100	25	−46 to 96
Benefit^b^	160	2328	931	159	2129	957	200	−14 to 413	87	−105 to 279
Component Costs at 15 month										
Health & social care excluding intervention^c^	152	4874	6317	149	4708	6383	166	−1577 to 1910	−231	−1734 to 1272
Charity^c^	152	63	215	149	49	230	14	−39 to 67	24	−37 to 84
Lost productivity^c^	152	4731	2674	149	3880	3027	850^a^	127 to 1573^a^	608	−25 to 1240
Patient^c^	152	24	141	149	30	162	−6	−38 to 27	−6	−37 to 25
Benefits^c^	152	1089	439	149	1049	441	40	−70 to 150	−24	−125 to 76
Intervention	213	316	173	193	4	0	312^a^	267 to 357^a^	3142^a^	268 to 359^a^
Total Costs at 15 months										
Health & social care including intervention^f^	152	5209	6326	149	4711	6383	498	−1248 to 2244	95	−1410 to 1599
Societal perspective including intervention^f^	152	11,116	7271	149	9720	7707	1396	−684 to 3476	675	−1039 to 2388

All figures are rounded to nearest whole number

^a^Confidence interval excludes zero

^b^Costs for a 6 month retrospective period

^c^Costs for a 3 month retrospective period

^d^Adjusting for clustering of care coordinator only

^e^Includes covariates for baseline: equivalent cost, SF-36 mental component score, SF-36 physical component score, EQ-5D-3 L utility, SF-36 utility, gender, ethnicity and borough, plus clustering for care coordinator

^f^Fifteen month costs discounted

Comparisons of total costs from both health and social care and societal perspectives at 15 months suggested no difference between the trial arms although the 95% confidence intervals suggest a tendency for societal costs to be greater in the intervention arm (Table 3). All sensitivity analyses confirmed this conclusion.

There were no differences in outcome at any of the assessments (Table 4). As with cost data, all sensitivity analyses confirmed this conclusion.

**Table 4:** Outcomes at baseline, 12 and 15 months (all 15 month outcomes discounted)

	Intervention n = 213			Control n = 193			Unadjusted mean difference^b^	95% CI^b^	Adjusted mean difference^c^	95% CI^c^
	*valid n*	*Mean*	*SD*	*valid n*	*Mean*	*SD*				
Baseline										
SF-36 mental component score	213	41.37	13.26	193	42.25	11.81	−0.88	−3.44 to 1.68	−0.26	−1.55 to 1.02
SF-36 physical component score	213	45.83	10.94	193	47.04	9.26	−1.20	−3.31 to 0.91	−0.60	−1.72 to 0.52
SF-36 utility	210	0.69	0.16	192	0.71	0.14	−0.02	−0.05 to 0.02	0.00	−0.01 to 0.01
EQ-5D-3 L utility	211	0.76	0.31	193	0.79	0.28	−0.02	−0.08 to 0.04	0.01	−0.04 to 0.06
12 months										
SF-36 mental component score	160	43.18	13.31	158	44.09	13.47	−0.91	−3.94 to 2.11	−0.05	−2.64 to 2.55
SF-36 physical component score	160	46.76	11.23	158	49.02	10.55	−2.27	−4.74 to 0.21	−1.45	−3.56 to 0.66
SF-36 utility	158	0.70	0.16	155	0.71	0.15	−0.02	−0.05 to 0.02	−0.00	−0.03 to 0.02
EQ-5D-3 L utility	159	0.80	0.25	156	0.80	0.28	0.00	−0.06 to 0.06	0.03	−0.03 to 0.08
15 months										
SF-36 mental component score	152	42.47	13.58	149	45.01	13.65	−2.54	−6.00 to 0.92	−0.80	−3.66 to 2.06
SF-36 physical component score	152	47.25	11.62	149	48.54	9.88	−1.29	−4.02 to 1.44	−0.68	−3.01 to 1.65
SF-36 utility	149	0.66	0.14	148	0.70	0.15	−0.03^a^	−0.07 to −0.00^a^	−0.02	−0.05 to 0.01
SF-36 based QALY gain	134	0.17	0.03	139	0.17	0.09	−0.01	−0.01 to 0.00	−0.00	−0.01 to 0.00
EQ-5D-3 L utility	152	0.77	0.24	149	0.80	0.25	−0.02	−0.09 to 0.04	0.00	−0.06 to 0.06
EQ-5D-3 L based QALY gain	137	0.19	0.05	140	0.20	0.06	−0.00	−0.02 to 0.01	0.00	−0.01 to 0.02

^a^Confidence interval excludes zero

^b^Adjusting for clustering of care coordinator

^c^Includes covariates for baseline: SF-36 mental component score, SF-36 physical component score, EQ-5D-3 L utility, SF-36 utility, gender, age, ethnicity, place of birth and borough, plus clustering for care coordinator

### Cost-effectiveness

**Fig. 1:**
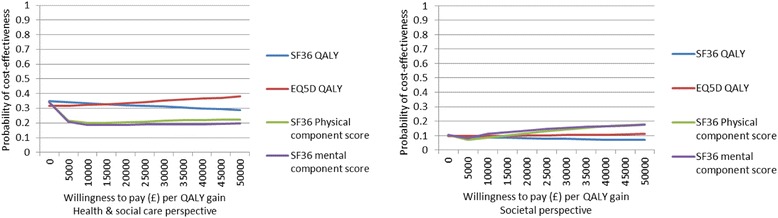
Cost-effectiveness acceptability curves for SF-36 physical and mental component scores plus SF-36 and EQ-5D-3 L based QALYs from a health & social care perspective and societal perspective

From a health and social care perspective, the probability of the IMPaCT Therapy being cost-effective does not exceed 0.4 for any of the examined willingness to pay thresholds for QALY gains (based on either the SF-36 or EQ-5D-3 L) or for the physical and mental component scores gains (Fig. 1). Similarly, the probability of cost-effectiveness from a societal perspective does not exceed 0.2 (Fig. 1).

## Discussion

We found no evidence of a clear difference in health and social care or societal costs between the two trial arms, in quality of life outcomes or cost-effectiveness as a result of delivering a comprehensive and integrated health promotion intervention to people with established psychosis. The corresponding outcome evaluation discusses the many possible explanations for lack of outcome effect and the same factors will likely have impacted on costs and cost-effectiveness since a significant factor was lack of successful implementation of the IMPaCT Therapy. Briefly, they include policy and practice steps towards greater parity between mental and physical health care which took place during the study may have improved the health of both groups, staff turn-over meant a sizable proportion of participants did not receive the intervention, and care co-ordinators implementing the intervention struggled to deliver the minimum dose.

### Strengths and limitations of the study

This study was a pragmatic trial based in five NHS mental health trusts. The intervention was specifically designed to be accessible to as many people as possible by being delivered by care coordinators as part as care as usual rather than requiring people to attend add-on or group appointments. However, there were also some methodological limitations. Data on resource use were collected by self-report. This makes it subject to participant recall bias. However, the approach was necessary in relation to strengths of the study design – our interest in the full range of formal services used by this group, given the mental and physical health focus here, and also in broader societal costs which are of particular relevance for a patient group whose health and care needs can have economic impacts upon multiple sectors of society. Even a narrower cost perspective would have been hindered by a lack of integration of relevant health and social care sector client records and a possible lack of comparability in record systems for all study sites. There is though evidence for the reliability of the self-report approach in similar populations [33, 34] and there is no reason to believe that any biases related to data collection would be imbalanced between the two trial arms, particularly since the CSRI was administered by blinded assessors.

A further limitation is we may have double-counted resource use associated with the IMPaCT Therapy. We collected this information separately from care coordinators, rather than from patient participants, to avoid unblinding the assessors conducting the participant interviews. Patients would anyway have found it difficult to separately report care related to the IMPaCT Therapy since it was designed to be integrated into usual care. However, this inevitably means that patient reports of contacts with their care coordinator include inputs associated with the intervention. While this may double-count absolute estimates of costs for the intervention arm, this would result in over-estimation and thus bias against, rather than for, the intervention arm.

There has been some discussion around the validity of the SF-36 and EQ-5D-3 L among study participants with mental health problems, especially those with schizophrenia and other psychoses [35]. Although the two measures are commonly used, and indeed recommended, for economic evaluation to inform policy-making in England, Brazier et al. [35] suggest that neither scale performs particularly well in these particular patient groups in terms of quantitative testing against psychometric criteria and that both have a limited coverage of domains identified as relevant by people with mental health problems. Thus, it is unclear whether the lack of QALY difference between the two trial arms reflects a lack of intervention effect or limitations associated with the measurement properties of these two health-related quality of life measures. However, given the lack of effect based on the SF-36 mental and physical component scores, and all other outcome measures, it is unlikely that there was a difference in QALYs that we have been unable to detect.

Although the intervention was conducted for 9 months, cost-effective analyses were conducted on the 12-15 month data. There could have been larger cost and outcome differences at 9 months (the end of intervention) which reduced over time thus no significant differences were seen at 12 and 15 months. However, this ensures the cost-effectiveness of the intervention could not be misrepresented if any outcome improvements or cost savings were subsequently not sustained even for 3 months.

Finally, the time horizon of the evaluation is likely to have been insufficient to identify all relevant outcomes for this patient group, particularly given the longer term nature of the impacts of physical health problems. However, it is unlikely that any effects of the intervention would transpire in the longer term if absent in the short term.

We used the human capital approach to valuing productivity loss rather than the friction cost method. While the human capital approach may over-estimate absolute values for lost productivity, such over-estimation will only impact the findings of the economic evaluation if productivity outcomes are different between the control and intervention groups, which does not appear to be the case here. Further, results from a societal perspective, which includes productivity losses, is consistent with results from a health and social care perspective.

### Comparison with previous research

While a number of studies have demonstrated effectiveness of interventions to address lifestyle factors in similar patient groups [35–38] few include an economic evaluation.

Verhaeghe et al. [13] investigated the cost-effectiveness of a health promotion targeting physical activity and healthy eating in people with mental illness using a Markov decision model. The intervention consisted of 10 weeks of psycho-educational and behavioural group-based sessions, group based exercise (weekly 30 min supervised walking sessions), and individual support from the mental health nurses. The authors reported an incremental cost-effectiveness ratio of Euro 27,096 per QALY in men and Euro 40,139 per QALY in women although this was very sensitive to modelling assumptions.

Meenan et al. [14] reported on a randomised controlled trial and economic evaluation of a lifestyle intervention designed to reduce weight among individuals with serious mental illnesses who were taking antipsychotic medications. The authors reported no significant change in EQ-5D scores but reported ICERs between $1623 to $2527 per kilogram reduced depending on which costs were included and which cohort of patients were included (completers versus intention to treat). The authors also reported ICERs from $467 to $727 per mg/dL reduced (fasting glucose) depending on which costs and cohort were used.

Both these studies thus suggest greater costs associated with intervening to produce improved outcomes in this population.

### Implications for policy

As reported by Gaughran et al. [16] a health promotion intervention targeting multiple risk factors has proved difficult to integrate into usual care for many contextual and pragmatic reasons. This leaves an unaddressed care gap that carries significant implications for both patient health and economic costs. An RCT of a similar intervention from Denmark, likewise failed to show a clinically significant effect [39]. Other studies show promise that interventions targeting specific issues [36, 38, 40] may be simpler to implement or more effective in improving physical outcomes. It would be vital to assess the resource and cost-effectiveness implications of such models since add-on services would present additional care costs in the short-term. Current financial pressures in the NHS mental health care suggest challenges in delivering new services whether through new funding or reallocation of existing budgets - hence our attempt to develop an intervention that can be provided pragmatically within existing patient contacts.

## Conclusions

We found no evidence that an integrated health promotion intervention for people with established psychosis improves outcomes or achieves savings in health and social care or societal costs. Given the long term economic implications of increased cardiovascular risk and premature mortality for this population, it is vital that other options for early intervention are developed and assessed for cost-effectiveness is given the multiple pressures on health and social care budgets now and in the foreseeable future.

## Abbreviations

CEAC: cost effectiveness acceptability curve
CEP: cost effectiveness plane
CMHT: community mental health teams
CPA: Care Approach Programme
CSRI: Client Service Receipt Inventory
HPI: health promotion intervention
ICER: incremental cost effectiveness ratio
IMPaCT: integrated health promotion intervention
PCA: prescription cost analysis
QALYs: quality adjusted life years
RCT: randomised controlled trial
TAU: treatment as usual
